# Magnesium Hydroxide as a Versatile Nanofiller for 3D-Printed PLA Bone Scaffolds

**DOI:** 10.3390/polym16020198

**Published:** 2024-01-09

**Authors:** Wang Guo, Wenlang Bu, Yufeng Mao, Enyu Wang, Yanjuan Yang, Chao Liu, Feng Guo, Huaming Mai, Hui You, Yu Long

**Affiliations:** 1 State Key Laboratory of Featured Metal Materials and Life-Cycle Safety for Composite Structures, Guangxi University, Nanning 530004, China; 2111301002@st.gxu.edu.cn (W.B.); 2211393019@st.gxu.edu.cn (Y.M.); 2211301064@st.gxu.edu.cn (E.W.); 2111301074@st.gxu.edu.cn (Y.Y.); 2111301033@st.gxu.edu.cn (C.L.); 2 Guangxi Key Laboratory of Manufacturing System and Advanced Manufacturing Technology, School of Mechanical Engineering, Guangxi University, Nanning 530004, China; 3 Guangxi Key Laboratory of Oral and Maxillofacial Rehabilitation and Reconstruction, Guangxi Medical University, Nanning 530021, China; ndgf@163.com (F.G.); huamingmai@163.com (H.M.); 4 Department of Oral and Maxillofacial Surgery, College of Stomatology, Guangxi Medical University, Nanning 530021, China

**Keywords:** fused deposition modeling (FDM), magnesium hydroxide (Mg(OH)_2_), polylactic acid (PLA), bone scaffold, degradation properties, biological properties, mechanical properties

## Abstract

Polylactic acid (PLA) has attracted much attention in bone tissue engineering due to its good biocompatibility and processability, but it still faces problems such as a slow degradation rate, acidic degradation product, weak biomineralization ability, and poor cell response, which limits its wider application in developing bone scaffolds. In this study, Mg(OH)_2_ nanoparticles were employed as a versatile nanofiller for developing PLA/Mg(OH)_2_ composite bone scaffolds using fused deposition modeling (FDM) 3D printing technology, and its mechanical, degradation, and biological properties were evaluated. The mechanical tests revealed that a 5 wt% addition of Mg(OH)_2_ improved the tensile and compressive strengths of the PLA scaffold by 20.50% and 63.97%, respectively. The soaking experiment in phosphate buffered solution (PBS) revealed that the alkaline degradation products of Mg(OH)_2_ neutralized the acidic degradation products of PLA, thus accelerating the degradation of PLA. The weight loss rate of the PLA/20Mg(OH)_2_ scaffold (15.40%) was significantly higher than that of PLA (0.15%) on day 28. Meanwhile, the composite scaffolds showed long-term Mg^2+^ release for more than 28 days. The simulated body fluid (SBF) immersion experiment indicated that Mg(OH)_2_ promoted the deposition of apatite and improved the biomineralization of PLA scaffolds. The cell culture of bone marrow mesenchymal stem cells (BMSCs) indicated that adding 5 wt% Mg(OH)_2_ effectively improved cell responses, including adhesion, proliferation, and osteogenic differentiation, due to the release of Mg^2+^. This study suggests that Mg(OH)_2_ can simultaneously address various issues related to polymer scaffolds, including degradation, mechanical properties, and cell interaction, having promising applications in tissue engineering.

## 1. Introduction

Bone scaffolds act as a temporary extracellular matrix and can provide a suitable physical, chemical, and biological microenvironment for cell growth and new bone regeneration. Therefore, they need to have a good degradation ability, biological activity, cell response, and suitable mechanical properties [1]. At present, there are many different types of materials applied in bone scaffolds including polymers, ceramics, and metals; they each have their own advantages and disadvantages [2,3,4]. Synthetic polymers are currently the most widely studied scaffold materials, especially biodegradable polymer materials [5]. PLA, a type of aliphatic polyester, has good biocompatibility, biodegradability, and processability. It can be completely degraded into carbon dioxide and water and then excreted through the body’s metabolism [6], thus being approved by the U.S. Food and Drug Administration (FDA) for clinical use [7]. Currently, PLA has been widely used in tissue engineering and drug delivery systems [8].

However, there were still some problems that hindered the application of PLA in bone tissue engineering. Firstly, the degradation rate of bone scaffolds should match the rate of new bone growth [9], but the degradation rate of PLA is much slower; its in vivo degradation lasts for more than four years [10], which is much slower than the regeneration rate of bone defects; it takes about several months to years [11]. In a word, a much slower degradation of PLA is unbeneficial to provide the adequate space for new bone formation. Secondly, the accumulation of acidic products produced by PLA degradation may lead to aseptic inflammatory reactions [12]. In terms of biological performance, PLA, similar to aliphatic polymers, faces the problems of a weak biological activity and cell response [13]. In addition, the mechanical properties of PLA need to be improved to provide a more stable mechanical support during bone regeneration and reconstruction [14].

To better apply PLA in developing bone scaffolds, much effort has been devoted to improving PLA. Polymer blending, which involves adding a polymer with a faster degradation rate, such as PGA [15] or PEG [16], is a common method to improve the degradation rate of PLA-based scaffolds. However, the accelerated degradation rate of the composite scaffold is primarily caused by the rapid degradation of the added polymer and does not fundamentally alter the degradation rate of PLA itself. Adding hydroxyapatite, the main inorganic component of bone, is one common method to improve the biomineralization ability of PLA bone scaffolds [17]. However, the biomineralization ability primarily depends on the speed of ion exchange. Since hydroxyapatite degrades slowly and has a slow ion exchange rate, its ability to enhance the biomineralization of PLA is limited. Loading bone-related growth factors, such as BMP [18] and VEGF [19], onto bone scaffolds is one common method to improve the cell response and promote new bone growth. However, as biological molecules, these growth factors have unstable physicochemical properties and are susceptible to inactivation and loss of effectiveness during loading and post-processing procedures [20].

Metal Mg, with a high reactivity, can degrade fast in an aqueous solution, producing magnesium hydroxide (MgOH_2_) and releasing Mg^2+^. Mg(OH)_2_ is alkaline and can be used to neutralize the acidic degradation products of polyester. The Mg element, an important macroelement widely existing in bone tissue, plays a significant role in bone metabolism and growth by affecting the cell activity of osteoblasts and osteoclasts through activating some pathways [21]. Thus, metal Mg has the potential to improve the degradation properties and cell responses of polyesters [22]. However, the degradation of pure Mg will release hydrogen gas, resulting in the formation of hydrogen gas pockets (Mg + 2H_2_O = Mg^2+^ + 2OH^−^ + H_2_↑) [23,24], which are harmful to bone growth. Additionally, the chemical activity and degradation rate of metal Mg are much higher; after being soaked in an aqueous solution for several days, it can completely degrade in only one to two weeks, which can lead to a significant pH change of the solution along with the generation of large quantities of hydrogen gas.

Mg(OH)_2_ is a basic hydroxide of magnesium that, unlike metallic Mg, does not undergo a hydrogen evolution reaction upon degradation. It can degrade and release Mg^2+^ and OH^−^ in a mild manner. If Mg(OH)_2_ was incorporated into polyesters, a sustained release of Mg^2+^ may be more beneficial for cell proliferation and osteogenic differentiation. On the other hand, the release of OH^−^ may accelerate the degradation rates of polyesters and neutralize the acid products. In addition, as a rigid inorganic material, Mg(OH)_2_ can act as a reinforcing filler to improve the mechanical properties of polymers [25]. Therefore, if we incorporate Mg(OH)_2_ nanoparticles into PLA, this is expected to simultaneously improve the degradation, biomineralization, cell response, and mechanical properties of the PLA.

The preparation process is another important factor in determining the performance of bone scaffolds besides the material design. Traditional preparation methods for bone scaffolds mainly include solvent casting [26], gas foaming [27], freeze-drying [28], phase separation [29], etc. It is difficult for these methods to build a complex, personalized bone scaffold and precisely control the porous structure. Three-dimensional printing technologies, based on the principle of discrete accumulation, could theoretically realize the precise forming of complex structures, which could accurately meet the controllable building of porous structures and personalized shapes for bone scaffolds [30,31]. Due to the wide range of processable materials, low material and use cost, environmental protection, and nontoxicity, fused deposition modeling (FDM), as the most common 3D printing technology, offers the potential for design and manufacturing in the combination of polymers and the biomedical field [32,33]. Furthermore, FDM has shown obvious competitiveness in the preparation of biodegradable polymer bone scaffolds including PLA [34,35,36]. However, to the best of our knowledge, there are few studies currently available on PLA/Mg(OH)_2_ porous bone scaffolds, let alone those using FDM 3D printing technology.

In this study, we incorporated Mg(OH)_2_ nanoparticles into PLA, used FDM 3D printing technology to prepare PLA/Mg(OH)_2_ composite bone scaffolds with various contents, and then investigated their comprehensive performances. The phase composition of PLA/Mg(OH)_2_ was detected using XRD and FTIR. The mechanical properties of PLA/Mg(OH)_2_ composite scaffolds were tested using tensile and compressive tests. The degradation and Mg^2+^ release behaviors of the composite scaffolds were evaluated using a PBS immersion test, and the degradation mechanism was discussed. The biomineralization of the PLA/Mg(OH)_2_ composite scaffolds was evaluated using the SBF immersion test. The cell response behaviors of BMSCs on the composite scaffolds were evaluated in the aspects of cell adhesion, proliferation, and differentiation.

## 2. Materials and Methods

### 2.1. Materials

The materials used in the experiments are mainly included: PLA (REVODE, Zhejiang Hisun Biomaterials Co., Ltd., Taizhou, China); Mg(OH)_2_ nanopowders (~200 nm, as shown in Figure S1) (Beijing Deke Science and Technology Co., Ltd., Beijing, China); dichloromethane (CH_2_Cl_2_, ≥99.5%, Sichuan Xilong Science Co., Ltd., Chengdu, China); simulated body fluid (SBF) (R24167, 1.5×, Shanghai Yuanye Bio-Technology Co., Ltd., Shanghai, China); rabbit bone marrow mesenchymal stem cells (BMSCs) (RBXMX-01001) and BMSC complete culture medium (RBXMX-80011) (Cyagen Biosciences, Inc., Guangzhou, China); DAPI staining solution (C1005), actin-tracker red-rhodamine (C2207S), BCIP/NBT alkaline phosphatase color development kit (C3206) (Shanghai Beyotime Biotechnology Co., Ltd., Shanghai, China); and phosphate buffered solution (Yida Technology Co., Ltd., Quanzhou, China).

### 2.2. 3D Printing of the PLA/Mg(OH)_2_ Scaffolds

The schematic diagram of the preparation process for the PLA/Mg(OH)_2_ composite filaments and scaffold is shown in Figure S2. Firstly, the PLA/Mg(OH)_2_ composites were prepared using a solvent mixing method in a fume hood: (1) PLA granules were dissolved in dichloromethane (DCM) (*w*/*v* = 1 g/10 mL) with magnetic stirring for 1 h at 40 °C; (2) Mg(OH)_2_ nanopowders were dispersed in DCM (*w*/*v* = 1 g/10 mL) using an ultrasonic dispersing treatment at room temperature for 15 min; (3) the Mg(OH)_2_ suspension was poured into the PLA solution, and then underwent magnetic stirring until most of the solvent DCM was volatilized; (4) the PLA/Mg(OH)_2_ mixed suspension was poured into a stainless steel plate and air-dried overnight for further solvent volatilizing and film formation; (5) the films were cut into fragments, and then dried in a drying oven at 55 °C for 24 h to reach constant weight; (6) finally, the fragments were used for subsequent filament preparation. The weight fractions of Mg(OH)_2_ in the composites were as follows: 0 wt%, 2.5 wt%, 5 wt%, 7.5 wt%, 10 wt%, and 20 wt%, and the corresponding composites were labeled as PLA, PLA/2.5Mg(OH)_2_, PLA/5Mg(OH)_2_, PLA/7.5Mg(OH)_2_, PLA/10Mg(OH)_2_, and PLA/20Mg(OH)_2_, respectively.

The PLA/Mg(OH)_2_ filaments were prepared using a single-screw extruder (Wellzoom C, Shenzhen Misida Technology Co., Ltd., Shenzhen, China). The temperatures of the extruding and plasticizing zones of the extruder were 165 °C and 170 °C, respectively. The filaments with a diameter of 1.75 ± 0.1 mm were obtained by adjusting the extrusion speed and pulling speed.

The PLA/Mg(OH)_2_ scaffold samples were prepared using an FDM 3D printer (End-er-3 S1 Pro, Shenzhen Creality 3D Technology Co., Ltd., Shenzhen, China). The 3D model of the PLA/Mg(OH)_2_ porous scaffold was designed using SolidWorks 2022, as shown in Figure S3. Considering that our research focuses on the effect of Mg(OH)_2_ content on the performance of composite scaffolds, we have kept the designed porosity constant at 50%. The main process parameters are set as follows: nozzle diameter = 0.4 mm (this size has a balance of printing accuracy, production rate, and printing stability according to pre-experiment), layer height = 0.2 mm, printing speed = 30 mm/s, nozzle temperature = 190 °C, hot bed temperature = 55 °C, and filling density = 100%. Other parameters are shown in Table S1.

### 2.3. Physical and Chemical Properties

The three-dimensional macroscopic appearance of the scaffolds was imaged with a digital camera; the top and side macroscopic appearances of the scaffolds were observed with an industrial camera (TD2KHU, Shenzhen Sanqiang Taida Optical Instrument Co., Ltd., Shenzhen, China); the microscopic appearance of the struts of the scaffolds was observed using SEM (SU8020, HITACHI, Tokyo, Japan).

The porosity of the PLA/Mg(OH)_2_ scaffold was determined according to the liquid displacement method [37]: (1) First, calculate the volume (*V*) based on the actual length, width, and height of the scaffold. (2) Place the scaffold in a 5 mL graduated cylinder, inject deionized water into the graduated cylinder until it reaches 4 mL using a syringe, and record the amount of water consumed (∆*V*) in the syringe at this point. (3) At this point, the volume of the solid part of the scaffold can be calculated as (4 − ∆*V*). Therefore, the actual porosity of the scaffold can be calculated using the following formula:

$$Porosity=\left(1-\frac{4-△V}{V}\;\right)\times 100\%$$

The functional groups of the raw Mg(OH)_2_ nanopowders and composite samples were measured using the Fourier transform infrared spectrometer (FTIR, iCAV9, Tianjin Energy Spectrum Technology Co., Ltd., Tianjin, China). The FTIR spectra were recorded using 32 scans at a resolution of 8 cm^−1^. The phase composition and crystal structure of PLA/Mg(OH)_2_ scaffolds were analyzed using XRD (D8, Bruker Co., Karlsruhe, Germany) with a scanning range of 2θ = 5–80° and a speed of 8°/min.

To evaluate the hydrophilicity of PLA/Mg(OH)_2_, the water contact angle (WCA) was measured using a contact angle measuring instrument (SDC-200S, Dongguan SINDIN Precision Instrument Co., Ltd., Dongguan, China). Firstly, 5 μL of distilled water was dropped on the composite samples which were prepared using an FDM printer (50 × 10 × 1 mm^3^), then the shape of the water drop was captured using the camera equipped with the measuring instrument, and finally, the water contact angle was measured and recorded.

### 2.4. Mechanical Properties

The mechanical properties of the PLA/Mg(OH)_2_ composites were tested using a universal material testing machine (AGS-X, Shimadzu, Kyoto, Japan), in terms of tensile and compressive properties. The tensile sample was 1BB type (referred to as GB-T 1040.2-2006), as shown in Figure S4; the loading speed was set at 2.5 mm/min. The compression test used 10 × 10 × 10 mm^3^ porous scaffolds with a pore and strut size of 800 μm and theoretical porosity of 50% (as shown in Figure S3), and the loading speed was set at 1 mm/min. The cross-sectional morphology of the PLA/Mg(OH)_2_ samples treated with liquid nitrogen fracture was observed using SEM.

### 2.5. Degradation Properties

The degradation properties and Mg^2+^ release behavior of the PLA/Mg(OH)_2_ scaffolds were evaluated using a PBS immersion test, and then the water absorption, mass loss, pH, and Mg^2+^ concentration were determined. The main experiment steps are as follows: (1) Each scaffold (10 × 10 × 3.2 mm^3^) was dried in a drying oven to a constant weight, and then the dry weight was recorded with an analytical balance (accuracy: 1.0 mg) as *m*_0_; (2) the dried samples were separately added into centrifugal tubes contained PBS solution, and incubated in the drying oven at 37.5 °C for 7, 14, and 28 days; (3) after the scheduled time, the scaffold samples and soaking solutions were separately collected; the surface water of the scaffold samples was absorbed with filter paper, and then their wet weight was recorded using an electronic balance (accuracy: 1.0 mg) as *M_t_*; the pH and Mg^2+^ concentration of the soaking solutions were measured using a digital pH meter and an inductively coupled plasma atomic emission spectrometer (ICP-AES) (ICP700T, Suzhou Booway Instrument Technology Co., Ltd., Suzhou, China), respectively; (4) the wet scaffold samples were dried in a drying oven to a constant weight, and then the dry weight was recorded with an analytical balance as *m_t_*. The mass loss and water absorption were calculated according to the following equations, respectively:

$$\mathrm{Mass}\;\mathrm{loss}\;(\%)=\frac{m_{0}-m_{t}}{m_{0}}\times 100\%$$

$$\mathrm{Water}\;\mathrm{absorption}\;(\%)=\frac{M_{t}-m_{t}}{m_{t}}\times 100\%$$

where *m*_0_ and *m_t_* are the dry weights of the scaffold samples before and after immersion, respectively; *M_t_* is the wet weight of scaffold samples after immersion.

Additionally, the surface morphology of the scaffold samples after immersion was observed using SEM, and the phase composition was analyzed with XRD using mapping mode.

### 2.6. Biomineralization

The biomineralization of the scaffolds (the ability to deposit hydroxyapatite) was evaluated by immersing the PLA/Mg(OH)_2_ scaffolds in SBF. First, the SBF powders were dissolved in 900 mL of deionized water at 37 °C, and the pH of the solution was titrated to 7.4 with dilute hydrochloric acid (2 mol/L). The scaffolds were weighed and put into a centrifuge tube, and the SBF was dripped into the centrifuge tube through a pipette gun (the ratio of the sample mass to the solution volume was *w*/*v* = 1 g/30 mL), and then we put the centrifuge tube into a vacuum oven at 37.5 °C for incubation. The scaffolds were taken out on days 7, 14, and 28 and then dried to a constant weight. The surface morphology of PLA and PLA/Mg(OH)_2_ scaffolds after the immersion test was observed using SEM. The deposition of apatite on the surface of the scaffolds was analyzed using XRD.

### 2.7. Cell Responses

The cell responses of PLA/Mg(OH)_2_ scaffolds were evaluated through cell culture of BMSCs in terms of cell adhesion, proliferation and osteogenic differentiation using fluorescence staining and ALP staining. The scaffold samples (10 × 10 × 1.6 mm^3^) were sterilized by ethylene oxide, and then immersed in PBS overnight for wetting. BMSCs were cultured in a complete medium and seeded on the scaffold samples in a 24-well plate, and then incubated at 37 °C for different times.

For the fluorescence staining, the scaffold samples were treated as follows after a scheduled time (1, 3, and 7 days): (1) gently washed with PBS 3 times for 5 min and then fixed by 4% formaldehyde solution in PBS; (2) added to 0.1% Triton X-100 in PBS to increase permeability; (3) stained with phalloidin solution; (4) stained with DAPI solution; and (5) finally, the scaffolds were observed and imaged with fluorescence microscopy to evaluate the cell proliferation.

For ALP staining, the scaffold samples were treated as follows: (1) after the cells adhered, the culture medium was replaced with an osteogenic induction medium containing vitamin C (0.2 mM), dexamethasone (10^−7^ M), and β-glycerophosphate (10 mM); (2) after the osteogenic induction time reached 7 and 14 days, the cells were stained with BCIP/NBT ALP staining solution; after incubating for 30 min, the staining solution was sucked off, and the color reaction was terminated by washing with triple distilled water; (3) finally, the stained cells were observed using an inverted phase-contrast microscope (Eclipse Ts2, Nikon, Tokyo, Japan).

### 2.8. Statistical Analysis

Quantitative data were expressed as the mean ± standard deviation. The Student’s *t*-test was performed to determine the statistical significance between groups, and it was considered to be significantly different when *p* < 0.05.

## 3. Results and Discussion

### 3.1. Morphology and Porosity of PLA/Mg(OH)_2_ Scaffolds

**Figure 1:**
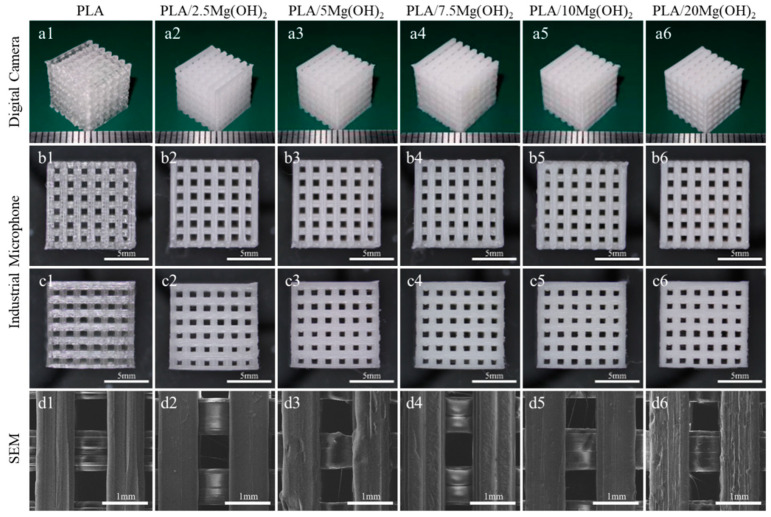
Morphology characterization of FDM 3D-printed PLA/Mg(OH)_2_ scaffolds: three-dimensional macroscopic appearance with digital camera (**a1**–**a6**); top view (**b1**–**b6**) and side view (**c1**–**c6**) with digital camera; the strut appearance with SEM (**d1**–**d6**). Notes: the prepared filaments have a good FDM printing quality, and the FDM 3D printed scaffolds show a well-ordered and interconnected three-dimensional porous structure.

The macroscopic and microscopic morphology of porous PLA/Mg(OH)_2_ scaffolds are shown in Figure 1. From the macroscopic morphology (Figure 1(a1–a6)), all the scaffolds showed a well-ordered and interconnected three-dimensional porous structure; the color of the scaffolds turned milky white after adding Mg(OH)_2_. From the top view (Figure 1(b1–b6)), the pore size was approximately 800 μm, and the strut size was also approximately 800 μm. From the side view (Figure 1(c1–c6)), the interlayer bonding between the extruding fibers was tight. The SEM images (Figure 1(d1–d6)) show that the strut is dense without an apparent defect, indicating a good printing quality; additionally, the surface of the struts became rough after adding Mg(OH)_2_, which might positively affect the hydrophilicity and cell growth of the scaffolds.

The actual porosities of all scaffolds are slightly lower than the designed theoretical porosity (~50%), as shown in Table S2. The reason is due to the expansion swelling effect, which is a common phenomenon in the melt extrusion of polymer materials [38]. In the FDM printing process, when the polymer material is extruded from the hot nozzle, the melt exhibits an expanded state, leading to an increase in the size of the extruded filament. The factors influencing the expansion swelling effect include the process parameters such as printing speed, extrusion temperature, nozzle diameter, as well as the rheological properties of the material [39]. It was known that the inorganic particles influenced the rheological properties of the polymer composites. An increase in the content of inorganic particles leads to an increase in the viscosity of composite materials [40]. In this study, when Mg(OH)_2_ particles were added to the PLA matrix, the viscosity of the melt increased. Ultimately, this leads to an increased expansion swelling of the composite filaments, resulting in an increase in the strut size and a decrease in the pore size and actual porosity of the scaffold.

### 3.2. Physical and Chemical Characterizations

**Figure 2:**
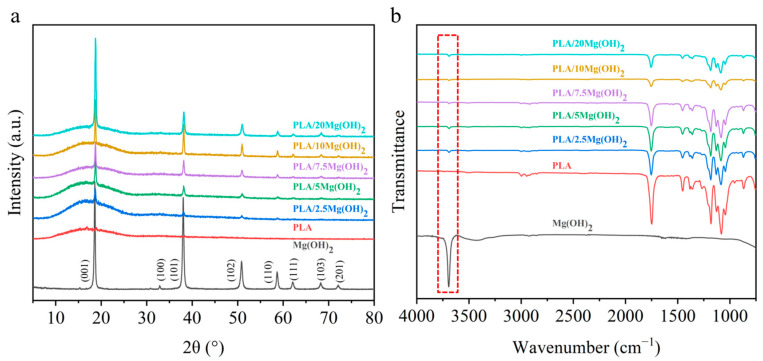
XRD patterns (**a**) and FTIR spectra (**b**) of PLA/Mg(OH)_2_. Notes: the results indicate Mg(OH)_2_ was composited with PLA without impurity formation after screw extrusion and FDM 3D printing.

The phase composition of the PLA/Mg(OH)_2_ scaffolds was analyzed using XRD (Figure 2a). The diffraction peaks of Mg(OH)_2_ were at 2θ = 18.6° (001), 32.8° (100), 38.0° (101), 50.8° (102), 58.7° (110), 62.2° (111), 68.2° (103), and 72.1° (201). PLA showed diffraction peaks at 16.9°. There appeared to be Mg(OH)_2_ peaks in the PLA/Mg(OH)_2_ scaffolds, and the peak intensity increased with the content. In addition, there were no obvious peaks of impurity. The XRD result indicated that Mg(OH)_2_ was successfully composited with the PLA, without the formation of impurities during the melt extrusion process [41].

The functional group structures of PLA/Mg(OH)_2_ composites were analyzed using FTIR, as shown in Figure 2b. Mg(OH)_2_ powders showed an obvious peak at 3691 cm^−1^ (the red dashed box labeled in Figure 2b) caused by an OH^−^ stretching vibration. Compared with the PLA, the peaks of Mg(OH)_2_ were observed in the PLA/Mg(OH)_2_ composites, and its peak intensity increased with the content. The presence of OH^−^ may enable Mg(OH)_2_ to form hydrogen bond interactions with PLA [42], which may positively affect the mechanical properties of PLA/Mg(OH)_2_ composites.

**Figure 3:**
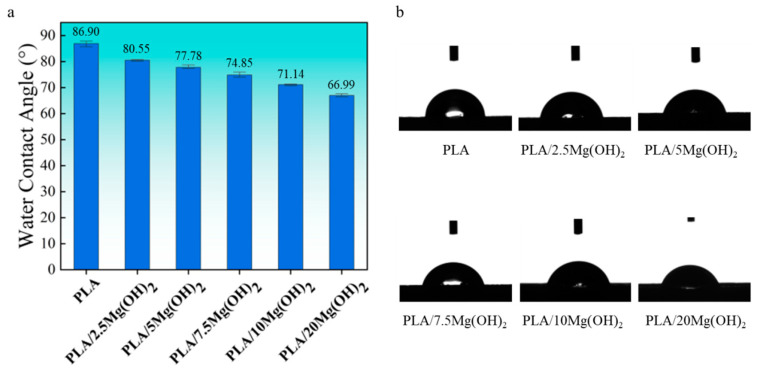
Water contact angle data (**a**) and images (**b**) of PLA/Mg(OH)_2_ composites. Notes: the hydrophilicity of PLA was effectively improved by Mg(OH)_2_.

Hydrophilicity is an essential property of biomaterials, which affects cell attachment, spread, and proliferation [43]. The hydrophilicity of PLA/Mg(OH)_2_ composites was evaluated using a water contact angle (WCA) test (Figure 3). PLA showed a WCA of 86.90°, located between hydrophilic and hydrophobic. The water contact angle gradually decreased with the content of Mg(OH)_2_. For PLA/20Mg(OH)_2_, the WCA decreased to 66.99°, which was 19.91° lower than PLA, indicating a significantly improved hydrophilicity. The results indicated that the hydrophilicity of PLA could be effectively improved by Mg(OH)_2_ and was closely related to the content.

### 3.3. Mechanical Properties

**Figure 4:**
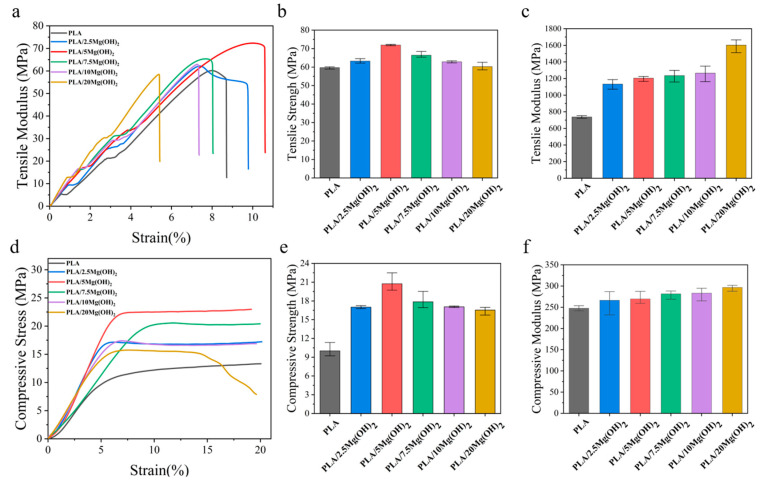
Mechanical properties of PLA/Mg(OH)_2_: typical tensile stress-strain curves (**a**), tensile strength (**b**), tensile modulus (**c**); typical compressive stress-strain curves (**d**), compressive strength (**e**), compressive modulus (**f**). Notes: as the content of Mg(OH)_2_ increased, the strength and compressive modulus of PLA/Mg(OH)_2_ increased first and then decreased, with an optimal content at 5 wt%.

Mechanical properties are significant for bone scaffolds as they provide mechanical support for bone defects after implantation [44]. The effect of Mg(OH)_2_ on the mechanical properties of PLA was evaluated using tensile and compression tests, as shown in Figure 4. The tensile strength of PLA/Mg(OH)_2_ composites increased gradually with the Mg(OH)_2_ content, reaching a peak value of 71.94 MPa at a 5 wt% content, which was significantly (*p* < 0.05) increased by 20.50% compared with PLA (59.72 MPa). However, when the content further increased to a 7.5–20 wt%, the tensile strength decreased instead. Nevertheless, the tensile strength of PLA/20Mg(OH)_2_ was still higher than that of PLA. The tensile modulus of PLA/Mg(OH)_2_ increased continuously with the content of Mg(OH)_2_ increasing, achieving a maximum value of 1602.71 MPa at a 20 wt%. The elongation at break increased first and then decreased with the content of Mg(OH)_2_ (Figure 4a), reaching a peak value at 5 wt%.

The compressive properties of PLA/Mg(OH)_2_ scaffolds are shown in Figure 4d–f. The compressive stress-strain curves could be divided into elastic and plastic stages (Figure 4d). In the elastic stage, the curves were approximately linear, with stress approximately proportional to the strain. During this stage, the scaffolds underwent an elastic deformation under compressive stress. As the compressive stress further increased, the curves entered the plastic stage, where the pore structure of the scaffold gradually compressed, and the scaffold gradually densified.

The compressive strength and modulus of the scaffolds were determined from the stress-strain curves, as shown in Figure 4e,f. The compressive strength of PLA/Mg(OH)_2_ scaffolds increased gradually with the content of Mg(OH)_2_ (Figure 4e), reaching a peak value of 19.66 MPa at a 5 wt%, which was significantly increased by 63.97% (*p* < 0.05) compared with PLA (11.99 MPa). However, with the content further increasing to a 7.5–20 wt%, the compressive strength decreased, but all the composite scaffolds were still stronger than PLA. The trend of the compressive modulus (Figure 4f) was similar to that of the compressive strength: as the content of Mg(OH)_2_ increased, the compressive modulus of PLA/Mg(OH)_2_ scaffolds increased to a peak value of 296.73 MPa at a 20 wt%, which was 19.87% higher than PLA (247.55 MPa). The mechanical test results demonstrated that Mg(OH)_2_ could significantly strengthen the PLA, and the strengthening effect was associated with the content. Natural bone can be classified into two types: cortical bone and trabecular (spongy) bone. Their mechanical strengths are approximately 100–200 MPa and 2–20 MPa, respectively [45]. In our results, the compressive strength of porous scaffolds ranged from 11.99 MPa to 19.66 MPa, meeting the strength requirements for spongy bone but showing a gap for cortical bone.

**Figure 5:**
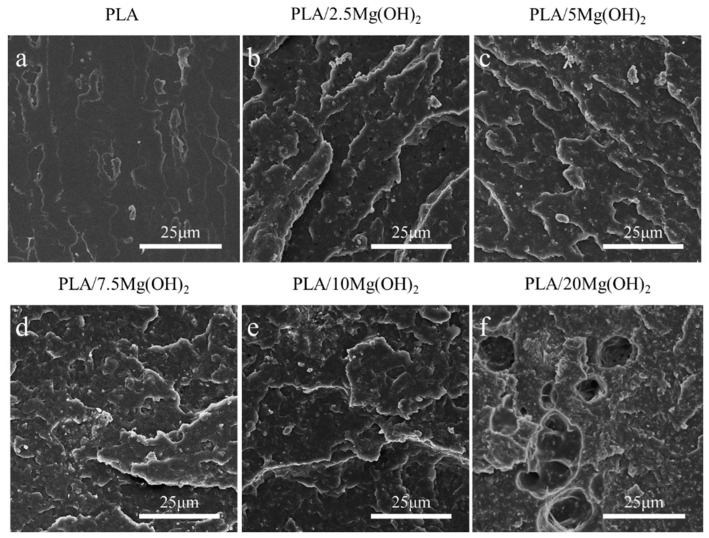
SEM images of liquid nitrogen fracture morphology for PLA/Mg(OH)_2_ composites: PLA (**a**), PLA/2.5Mg(OH)_2_ (**b**), PLA/5Mg(OH)_2_ (**c**), PLA/7.5Mg(OH)_2_ (**d**), PLA/10Mg(OH)_2_ (**e**), PLA/20Mg(OH)_2_ (**f**). Notes: at a low content (2.5 and 5 wt%), Mg(OH)_2_ particles can disperse relatively uniformly, thus exerting well mechanical strengthening effects; while at a high content (7.5–20 wt%), Mg(OH)_2_ particles tend to form obvious aggregates with poor bonding with PLA matrix.

The liquid nitrogen fracture morphology of the scaffolds was observed using SEM (Figure 5). The fracture surface of pure PLA was smooth without obvious deformation (Figure 5a), indicating a brittle fracture behavior. After adding Mg(OH)_2_ (Figure 5b,c), the fracture morphology of the PLA matrix became rough. This result indicated that Mg(OH)_2_ could strengthen the PLA matrix. When the content of Mg(OH)_2_ was added at relatively low levels (2.5 wt% and 5 wt%, Figure 5b,c), the dispersion of Mg(OH)_2_ nanoparticles in the PLA matrix was relatively uniform, and their interfacial adhesion with the PLA matrix was relatively good, which played a crucial role in the enhancement of the mechanical properties. However, when the Mg(OH)_2_ was added at excessive levels (7.5–20 wt% in this study, Figure 5d–f), the dispersion of the nanoparticles began to deteriorate, with large numbers of nanoparticles agglomerating together, leading to large amounts of defects and stress concentration [46]. As a result, the mechanical enhancing effect of Mg(OH)_2_ nanoparticles was weakened.

The content and distribution of nanofillers have significant effects on the mechanical properties of polymer nanocomposites [47]. At an appropriate content (no more than a 5 wt% in this study), Mg(OH)_2_ nanoparticles can be relatively dispersed in the PLA matrix and act as rigid phases; meanwhile, Mg(OH)_2_ may form good interfacial adhesion with PLA due to the hydrogen bond interaction [48], thus effectively absorbing stress from the relatively soft PLA matrix. As a result, the resistance to deformation and fracture of the matrix was strengthened, contributing to significant increases in the mechanical properties of PLA/Mg(OH)_2_. However, when at a relatively high content, Mg(OH)_2_ nanoparticles tend to form much agglomeration, thus weakening the mechanical strengthening effects.

Considering that the mechanical performance is optimal at a Mg(OH)_2_ content of 5 wt%, we have chosen to proceed with subsequent degradation, biomineralization, and cell culture experiments using this content. Additionally, to explore the influence of Mg(OH)_2_ content on PLA/Mg(OH)_2_, we have also included a 20 wt% content as a control in subsequent experiments.

### 3.4. Degradation Properties

**Figure 6:**
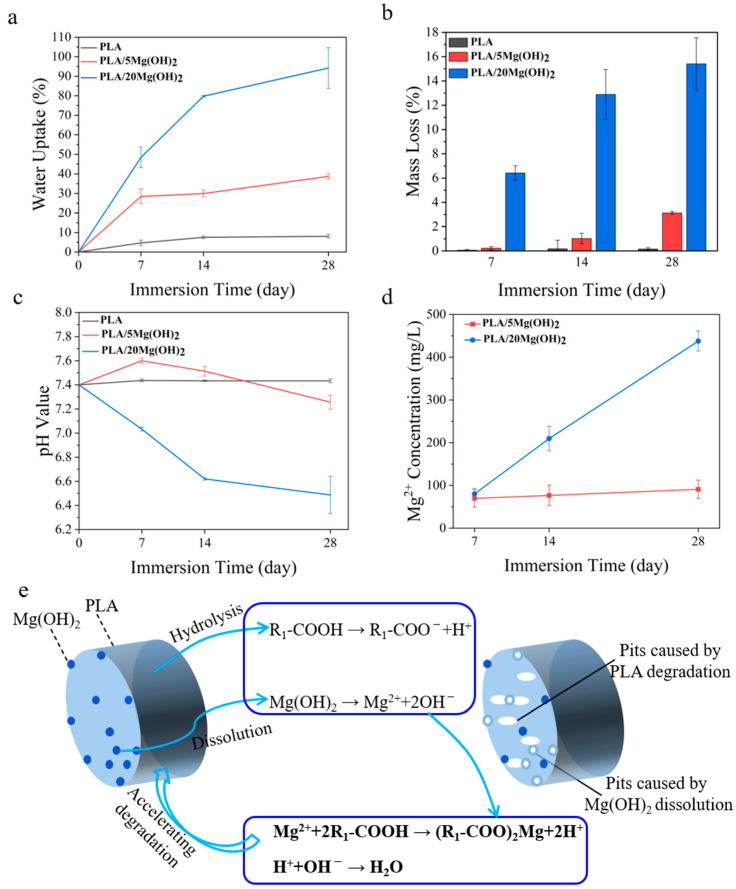
The degradation properties of PLA/Mg(OH)_2_: water absorption (**a**), mass loss (**b**), pH value (**c**), Mg^2+^ release concentration from scaffolds (**d**), the schematic diagram illustrating the mechanism by which Mg(OH)_2_ accelerated the degradation of PLA scaffolds (**e**). Notes: Mg(OH)_2_ accelerated the degradation of PLA by adjusting the acidity and alkalinity in the aqueous environment.

The degradation properties and Mg^2+^ release behavior of PLA/Mg(OH)_2_ scaffolds were tested with PBS immersion for 7, 14, and 28 days. The results of the water absorption rate, mass loss rate, pH value, and Mg^2+^ concentration are shown in Figure 6. The water absorption rate gradually increased with immersion time for all the scaffolds (Figure 6a). More importantly, the water absorption rate of PLA/Mg(OH)_2_ scaffolds was significantly higher than that of PLA at each time point, and it increased with the Mg(OH)_2_ content. On day 28, the water absorption rate of the PLA/20Mg(OH)_2_ scaffold reached 94.19%, which was significantly higher than that of the PLA scaffold (8.06%) (*p* < 0.05). For bone scaffolds, a high water absorption capacity is usually beneficial for nutrient delivery and cell affinity, thus helping to maintain metabolism and promoting cell proliferation [49]. Additionally, a high water absorption capacity could promote the degradation of PLA, which is more conducive to the growth of bone tissues.

The mass loss of PLA/Mg(OH)_2_ scaffolds is shown in Figure 6b. During the 28-day PBS immersion, the mass of the PLA scaffolds hardly changed. By contrast, PLA/Mg(OH)_2_ scaffolds showed an obvious mass loss, which was positively associated with the immersion time and Mg(OH)_2_ content. After 28 days of degradation, the mass loss rates of PLA, PLA/5Mg(OH)_2_, and PLA/20Mg(OH)_2_ scaffolds were 0.15%, 3.12%, and 15.40%, respectively.

The pH value change of the degradation medium PBS is shown in Figure 6c. During the 28-day PBS immersion, the pH value of the PLA scaffolds fluctuated little and remained almost unchanged at a pH = 7.4, indicating that almost no signs of degradation of the PLA occurred. The pH value of the PLA/5Mg(OH)_2_ scaffold showed a trend of increasing slightly at first and then decreasing, but remained nearly neutral at a pH = 7.4. However, the pH value of the PLA/20Mg(OH)_2_ scaffold continually decreased gradually with the immersion time, reaching 6.49 on day 28. The results of mass loss and pH indicated that Mg(OH)_2_ could effectively promote the degradation of PLA.

The Mg^2+^ release behavior of PLA/Mg(OH)_2_ scaffolds is shown in Figure 6d. As the degradation time prolonged, the Mg^2+^ concentration released from the scaffolds gradually increased. The PLA/20Mg(OH)_2_ scaffold released a higher amount of Mg^2+^ than the PLA/5Mg(OH)_2_ at each period.

The mechanism by which Mg(OH)_2_ promoted the degradation of PLA scaffolds is schematically illustrated in Figure 6e. In the PBS immersion, under the action of water molecules, the ester bonds of polylactic acid are cleaved to produce hydrolysis products with carboxyl and hydroxyl end groups (Equation (1)):
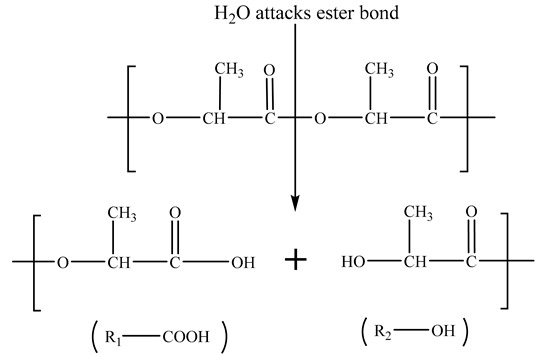
(1)

Equation (2) refers to the further dissociation of carboxyl groups that produce H^+^ ions, which lower the pH value in the environment. This has a “self-catalytic” effect on the degradation of PLA. Meanwhile, it promotes the dissolution of alkaline Mg(OH)_2_ in the solution, releasing Mg^2+^ and OH^−^ ions, as described in Equation (3).

R_1_-COOH→R_1_-COO^−^ + H^+^ (2)

Mg(OH)_2_ = Mg^2+^ + 2OH^−^ (3)

In return, the two types of products can consume the hydrolysis products of PLA (Equation (4)) and neutralize the acidic products of PLA (Equation (5)). This acid-base neutralization reaction continuously promotes the degradation of the PLA matrix. Why was the pH of the PLA/5Mg(OH)_2_ scaffold > 7.4 before 7 days, while the pH of the PLA/20Mg(OH)_2_ scaffold was < 7.4? The possible reasons can be explained as follows: before 7 days, the PLA/5Mg(OH)_2_ scaffold, due to its small degradation amount, had little catalytic degradation effect on the PLA, resulting in PLA with almost no degradation, which meant the acidic effect caused by the PLA degradation was still less than the alkaline effect caused by the degradation of Mg(OH)_2_ itself. Therefore, the pH remained alkaline before 7 days. In contrast, in the PLA/20Mg(OH)_2_ scaffold, there was a significant degradation of Mg(OH)_2_, leading to a substantial catalytic effect on the PLA degradation, even resulting in the acidic effect caused by the PLA degradation exceeding the alkaline effect caused by the degradation of Mg(OH)_2_ itself. As a result, the pH became acidic before 7 days. After 7 days, as the degradation of Mg(OH)_2_ continued, the catalytic effect of both the 5 wt% and 20 wt% scaffolds on PLA increased significantly, accelerating the degradation of the PLA. Thereby, the acidic effect produced by the PLA degradation surpasses the alkaline effect of Mg(OH)_2_ degradation, resulting in a pH < 7.4 after 7 days. The pH change trends indicated that Mg(OH)_2_ particles could accelerate the degradation of PLA by adjusting the acidity and alkalinity in the aqueous environment.

Mg^2+^ + 2R_1_-COOH→(R_1_-COO)_2_Mg + 2H^+^ (4)

H^+^+OH^−^→H_2_O (5)

**Figure 7:**
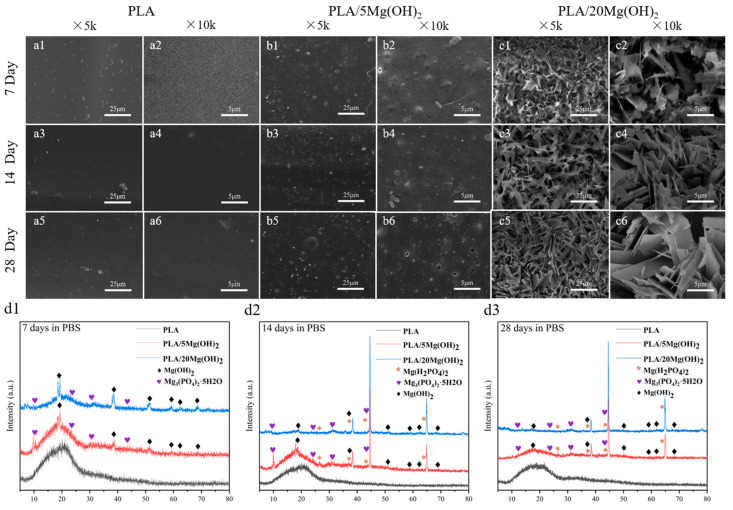
Degradation morphology and product analysis of PLA/Mg(OH)_2_ scaffolds after immersing in PBS for 7, 14, and 28 days. SEM morphology of PLA (**a1**–**a6**); PLA/5Mg(OH)_2_ (**b1**–**b6**); PLA/20Mg(OH)_2_ (**c1**–**c6**); XRD patterns after degradation (**d1**–**d3**). Notes: With the increase in Mg(OH)_2_ content and experimental time, the mixed crystalline whisker-like substance of magnesium dihydrogen phosphate and magnesium hydroxide appeared on the surface of the scaffolds.

The surface morphology of PLA/Mg(OH)_2_ scaffolds after 7, 14, and 28-day PBS immersion was observed using SEM, as shown in Figure 7a–c. For the PLA scaffold, during the whole 28-day immersion period, its surface remained smooth and flat, with almost no cracks and pits. For PLA/Mg(OH)_2_ scaffolds, during the initial 14 days, there were some white particles exposed on the surface of the PLA/5Mg(OH)_2_ scaffold (Figure 7(b1–b4)). According to the XRD analysis (Figure 7(d1)), the main components were Mg(OH)_2_ and Mg_3_(PO_4_)_2_·5H_2_O. The detachment of Mg(OH)_2_ and magnesium salts from the PLA matrix during degradation may be responsible for the formation of cracks and pits on the surface. For the PLA/20Mg(OH)_2_ scaffold (Figure 7(c1–c6)), there appeared to be whisker-like mixtures after only 7 days. According to the XRD analysis (Figure 7(d2)), the mixtures mainly consisted of magnesium salts (Mg_3_(PO_4_)_2_·5H_2_O and Mg(H_2_PO_4_)_2_) and Mg(OH)_2_. The deposits may be generated by the reaction between the Mg^2+^ released from Mg(OH)_2_ and the PO_4_^3−^ and HPO_4_^2−^ in the PBS solution. The results showed that Mg(OH)_2_ significantly improved the degradation performance of PLA.

### 3.5. Biomineralization

**Figure 8:**
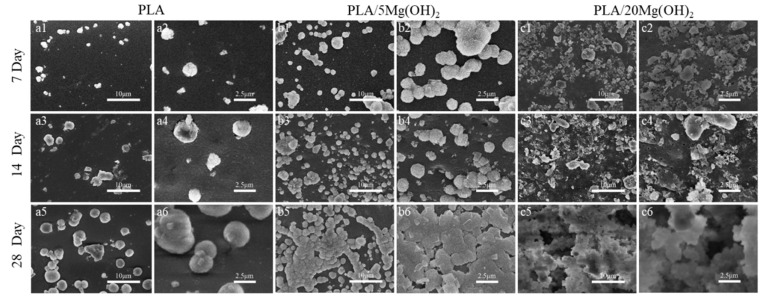
SEM surface morphology of PLA/Mg(OH)_2_ after immersion in SBF for 7, 14, and 28 days. SEM morphology of PLA (**a1**–**a6**); PLA/5Mg(OH)_2_ (**b1**–**b6**); PLA/20Mg(OH)_2_ (**c1**–**c6**). Notes: With the increase in Mg(OH)_2_ content and immersion time, the quantity and volume of white spherical mineral deposits on the surface of the scaffold gradually increased.

The biomineralization ability of PLA/Mg(OH)_2_ scaffolds was investigated using an SBF immersion for 7, 14, and 28 days, and the surface morphology was observed using SEM (Figure 8). There was only a small amount of white, spherical mineral deposits on the surface of the PLA scaffold during the immersion (Figure 8(a1–a6)); for the PLA/5Mg(OH)_2_ scaffold (Figure 8b), there were more spherical mineral deposits which increased in number and volume with the immersion time. On day 28, they were particularly tightly combined, with a finely textured cauliflower-shaped appearance that was observed using high-magnification SEM (Figure 8(b6)). On the PLA/20Mg(OH)_2_ scaffold (Figure 8c), lots of spherical mineral deposits could be observed after only 7 days (Figure 8(c1,c2)). As the immersion time was prolonged to 14 days, the number and density of the deposits increased, and the volume became larger with small white minerals constantly nucleating and growing on the large deposits, showing a phenomenon of multi-layer deposition (Figure 8(c3,c4)). On day 28, there were white deposits of different sizes growing together and overlapping layer by layer, becoming thick and dense (Figure 8(c5,c6)).

**Figure 9:**
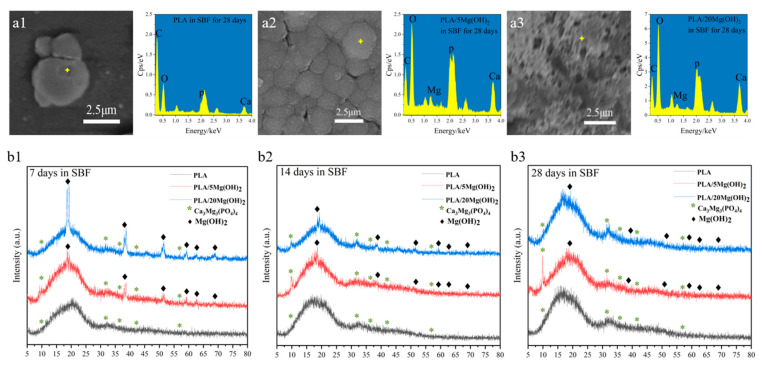
Element and phase analysis of mineral deposits on PLA/Mg(OH)_2_ after immersion in SBF for 7, 14, and 28 days: EDS spectra (**a1**–**a3**) and XRD patterns (**b1**–**b3**). Notes: EDS indicated the mineral deposits consisted mainly of Ca, P, and Mg elements, and XRD further indicated the mineral deposits consisted mainly of Ca_3_Mg_3_(PO_4_)_4_ (Mg-substituted apatite).

The elemental analysis of the white spherical deposits was performed using EDS (Figure 9(a1–a3)). The EDS spectra showed that the inorganic elements were mainly Ca and P, indicating that the deposits were mainly minerals containing Ca and P; a small amount of Mg was also detected due to the dissolution of Mg(OH)_2_. The XRD analysis (Figure 9(b1–b3)) further demonstrated that the deposits were mainly composed of calcium magnesium phosphate (Ca_3_Mg_3_(PO_4_)_4_, Mg-substituted apatite). With the increase in the immersion time, the peaks at 2θ = 10.50° and 31.70° of Mg-substituted apatite (Ca_3_Mg_3_(PO_4_)_4_) indicated further deposition and growth. Additionally, the XRD patterns indicated that the amount of Mg-substituted apatite increased with the content of Mg(OH)_2_. In addition, the EDS spectra also showed that the peak intensity of Ca and P elements were positively related to the Mg(OH)_2_ content (Figure 9(a3)). This may be attributed to Mg(OH)_2_ and Mg^2+^ as sites for the nucleation of apatite. The apatite plays significant roles in stimulating cell metabolism, bone regeneration, and chemical bonding between the biomaterial and bone tissues [50,51]. In a word, the results demonstrated that adding Mg(OH)_2_ could effectively improve the apatite-forming ability and biomineralization of PLA scaffolds. Based on the above experimental results, the PLA/5Mg(OH)_2_ scaffold with the best mechanical properties and the PLA/20Mg(OH)_2_ with the best degradation performance and biomineralization were selected for subsequent cell culture experiments.

### 3.6. Cell Responses

**Figure 10:**
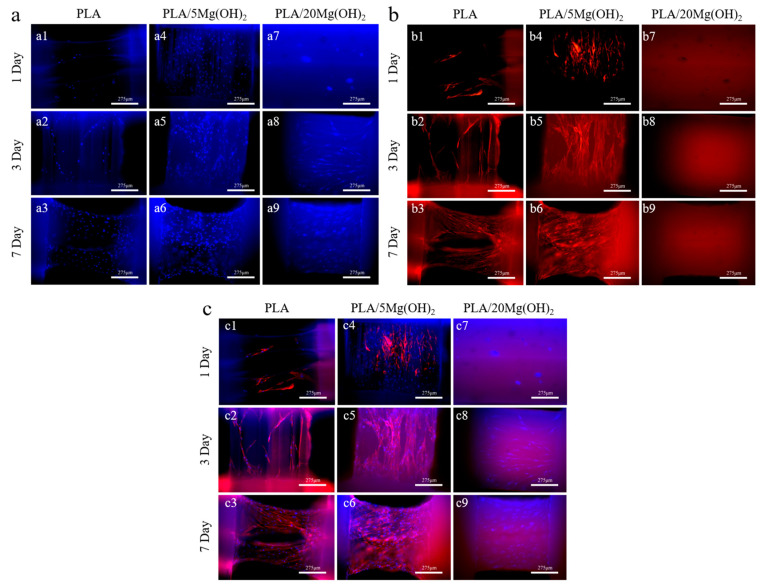
Fluorescence staining images of PLA and PLA/Mg(OH)_2_ scaffolds: DAPI (**a1**–**a9**), phalloidin (**b1**–**b9**), merged (**c1**–**c9**). Notes: Observations on the struts of the composite scaffold reveal a greater number of cell nuclei. The strut with PLA/5Mg(OH)_2_ shows the highest density of spread-out actin, while no actin is observed in PLA/20Mg(OH)_2_, indicating that the appropriate addition of Mg(OH)_2_ promotes cell proliferation and growth.

BMSCs were used to evaluate the cell response behaviors on PLA/Mg(OH)_2_ scaffolds. The fluorescence staining images after 1, 3, and 7 days of cell culture are shown in Figure 10. It was obvious that the number of cells on the PLA/5Mg(OH)_2_ scaffold (Figure 10(a4–a6)) was more than that on the PLA at each time point, with a better cell spreading morphology (Figure 10(b4–b6)). On day 7, the cells covered most of the strut (Figure 10(c6)). There was a “strange” phenomenon where the phalloidin staining (cell actin) was abnormal for the PLA/20Mg(OH)_2_ scaffold (Figure 10(b7–b9)), which may have resulted from the damage of excessive Mg(OH)_2_ and alkalinity on the staining reaction and cells. The results showed that an appropriate content of Mg(OH)_2_ was beneficial to enhance cell adhesion and proliferation.

**Figure 11:**
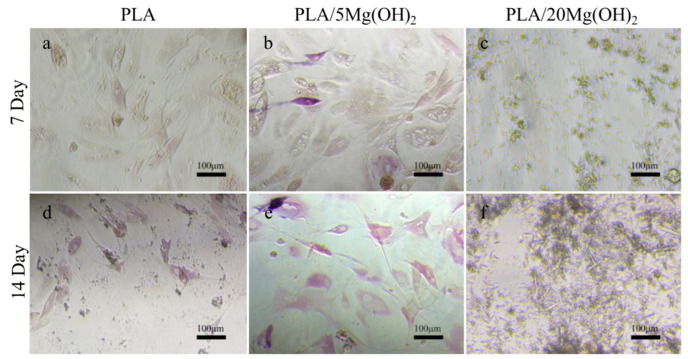
ALP staining images of BMSCs after culturing for 7 and 14 days: PLA (**a**,**d**), PLA/5Mg(OH)_2_ (**b**,**e**), PLA/20Mg(OH)_2_ (**c**,**f**). Notes: More cells with a higher degree of staining are observed in PLA/5Mg(OH)_2_, but only a large amount of whisker-like substances are seen in PLA/20Mg(OH)_2_, indicating that the appropriate addition of Mg(OH)_2_ is beneficial for enhancing the osteogenic differentiation ability of cells.

Alkaline phosphatase (ALP) is an enzyme responsible for the hydrolysis of phosphate esters into organophosphates, which is an early marker of osteoblast differentiation and bone matrix formation. BMSCs were used to evaluate the osteogenic differentiation using ALP staining (Figure 11). Compared with the PLA group (Figure 11a,d), the PLA/5Mg(OH)_2_ group (Figure 11b,e) showed a more positive ALP staining, and the stained cells increased with the culture time. This result indicated that Mg(OH)_2_ could improve the osteogenic differentiation of BMSCs. However, in the PLA/20Mg(OH)_2_ group (Figure 11c,f), there were no signs of cells, with a large amount of whisker-like substances appearing, which may have resulted from the precipitation of magnesium salts produced by the dissolution of excessive amounts of Mg(OH)_2_. As a result, the pH of the culture medium significantly changed, and the cell growth was hindered.

A neutral or weakly alkaline microenvironment is more suitable for cell growth, while neither acid nor alkaline conditions are favorable [52]. When Mg(OH)_2_ is added at an appropriate content (5 wt%), its dissolution can create a weakly alkaline microenvironment and release a moderate amount of Mg^2+^, thus promoting the cell response. However, when the addition of Mg(OH)_2_ was excessive (20 wt% in this study), the dissolution of large amounts of Mg(OH)_2_ led to a relatively higher alkalinity. The abnormal phenomenon of phalloidin staining in PLA/20Mg(OH)_2_ demonstrated the alkalinity was excessively high and was harmful to the cells.

It was reported that Mg^2+^ could regulate the expression of genes related to cell proliferation and osteogenic differentiation, including ALP, Col-1, Runx2, OCN, OPN, etc. [53,54]. In this study, PLA/5Mg(OH)_2_ can release Mg^2+^ into the culture medium without remarkably changing the pH, thus positively involving the cell metabolism and stimulating better cell adhesion, proliferation, and osteogenic differentiation. However, in PLA/20Mg(OH)_2_, the dissolution of excessive amounts of Mg(OH)_2_ resulted in a significant pH change in the culture medium, which negatively affected the cell responses and even prevailed over the positive stimulation of Mg^2+^ release. In summary, an appropriate content of Mg(OH)_2_ (~5 wt% in this study) can effectively improve the cell responses of PLA scaffolds.

Based on the experimental results above, it was indicated that Mg(OH)_2_ can simultaneously address various issues related to the PLA scaffold, including degradation, mechanical, and biological properties. The PLA/Mg(OH)_2_ scaffold with good comprehensive properties may have the potential to fill and repair bone defects in clinic. While promoting bone tissue regeneration, they gradually degrade, making space available for new bone regeneration. This can reduce the need for autografts and allografts, avoiding the disadvantages of multiple surgeries, limited donor availability, complications at the donor site, and immune rejection.

However, the current study also has some limitations. The following issues are worth exploring in future research: (1) Investigate the effects of different Mg(OH)_2_ particle sizes and shapes on the properties of the PLA/Mg(OH)_2_ scaffolds; (2) Explore the effects of more different contents of Mg(OH)_2_ on the biocompatibility and biodegradation of the PLA/Mg(OH)_2_ scaffolds; (3) Evaluate the long-term degradation and stability of the PLA/Mg(OH)_2_ scaffolds in vivo.

## 4. Conclusions

In this study, we prepared PLA/Mg(OH)_2_ composite bone scaffolds using FDM technology and investigated their comprehensive performances. The results showed that adding Mg(OH)_2_ improved the mechanical, degradation, and biological properties of PLA: (1) The addition of 5 wt% Mg(OH)_2_ resulted in optimal mechanical properties, with a 63.97% increase in the compressive strength and a 20.50% increase in the tensile strength compared to PLA. (2) Mg(OH)_2_ remarkably accelerated the degradation rate of PLA through an acid–base neutralization reaction; the mass loss of the scaffold after 28 days of immersion was increased from 0.15% to 15.40% by the addition of 20 wt% Mg(OH)_2_. (3) The composite scaffolds showed a long-lasting Mg^2+^ release of more than 28 days due to the encapsulation effects of the PLA matrix on the Mg(OH)_2_ nanoparticles. (4) Mg(OH)_2_ improved the nucleation and growth of apatite and enhanced the biomineralization ability of PLA scaffolds. (5) PLA/5Mg(OH)_2_ exhibited better cell adhesion, proliferation, and osteogenic differentiation of BMSCs due to Mg^2+^ release and a suitable pH microenvironment. The above research results showed that the Mg(OH)_2_ could be used as a multifunctional filler to enhance the mechanical, degradation, and biological properties of polymer bone scaffolds.

## Data Availability

Data will be made available on request.

## Supplementary Materials

The following supporting information can be downloaded at: https://www.mdpi.com/article/10.3390/polym16020198/s1, Table S1. FDM 3D printing parameters. Table S2 Actual porosity data of the PLA/Mg(OH)_2_ scaffolds (determined according to the liquid displacement method). Figure S1. Representative SEM morphology of Mg(OH)_2_. Figure S2. The schematic diagram of the preparation process for PLA/Mg(OH)_2_ composite, filament and scaffold. Figure S3. Schematic diagram of sample for the compression test. Figure S4. Schematic diagram of 1BB-type sample for the tensile test.
