# Supplementary material for: Magnesium Hydroxide as a Versatile Nanofiller for 3D-Printed PLA Bone Scaffolds
Source: Polymers (Basel). 2024 Jan 9;16(2):198. doi: 10.3390/polym16020198 (PMC10820754; doi:10.3390/polym16020198)
Supplement: Supplementary file 1 [file polymers-16-00198-s001.zip › polymers-2772563-supplementary.pdf]

polymers

Research Article

Magnesium Hydroxide as a Versatile Nanofiller for 3D Printed PLA Bone Scaffolds

Wang Guo [1,2,*], Wenlang Bu [1,2], Yufeng Mao [1,2], Enyu Wang [1,2], Yanjuan Yang [1,2], Chao Liu [1,2], Feng Guo [3,4], Huaming Mai [3,4], Hui You [1,2,*] and Yu Long [1,2,*]

1 State Key Laboratory of Featured Metal Materials and Life-cycle Safety for Composite Structures, Guangxi University, Nanning 530004, China
2 Guangxi Key Laboratory of Manufacturing System and Advanced Manufacturing Technology, School of Mechanical Engineering, Guangxi University, Nanning 530004, China
3 Guangxi Key Laboratory of Oral and Maxillofacial Rehabilitation and Reconstruction, Guangxi Medical University, Nanning 530021, China
4 Department of Oral and Maxillofacial Surgery, College of Stomatology, Guangxi Medical University, Nanning 530021, China
* Correspondence: guowang@gxu.edu.cn or drwangguo@outlook.com (W.G.); hyou@gxu.edu.cn or usmlhy@iim.ac.cn (Y.H.); longyu@gxu.edu.cn (Y.L.)

Supplementary Materials

Table S1. FDM 3D printing parameters.

Initial Layer Height	0.2mm
Wall Thickness	0.8 mm
Wall line count	2
Top/Bottom Thickness	0.0 mm
Top/Bottom Pattern	Lines
Top/Bottom Directions	[90°, 180°]
Infill Line Distance	0.8 mm
Infill Pattern	Grid
Build Plate Adhesion Type	Skirt
Skirt Line Count	1
Enable Retraction	√
Retraction Distance	6.5 mm
Retraction Speed	25.0 mm/s
Filling Density	100%

Table S2. Actual porosity data of the PLA/$Mg(OH)_2$ scaffolds (determined according to the liquid displacement method).

Samples	PLA	2.5$Mg(OH)_2$	5$Mg(OH)_2$	7.5$Mg(OH)_2$	10$Mg(OH)_2$	20$Mg(OH)_2$
Porosity (%)	48.57± 3.40	45.77± 2.64	47.31± 2.85	46.70± 2.37	44.53± 1.95	43.63± 0.78

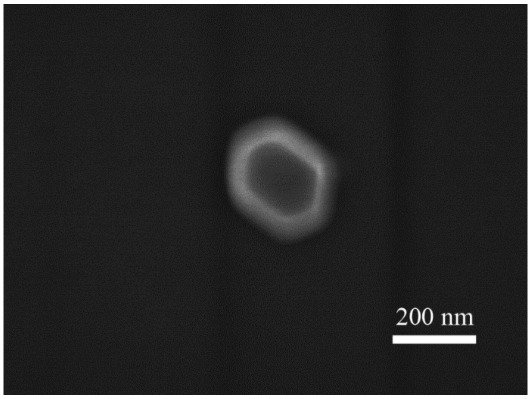

Figure S1. Representative SEM morphology of $Mg(OH)_2$.

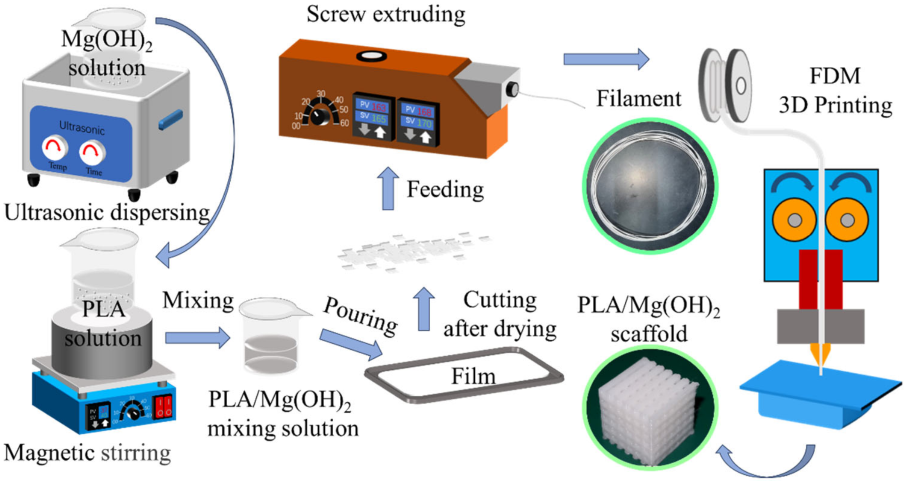

Figure S2. The schematic diagram of the preparation process for PLA/$Mg(OH)_2$ composite, filament and scaffold.

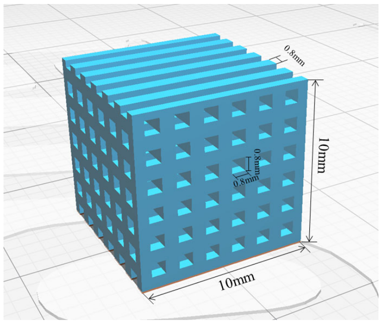

Figure S3. Schematic diagram of sample for the compression test.

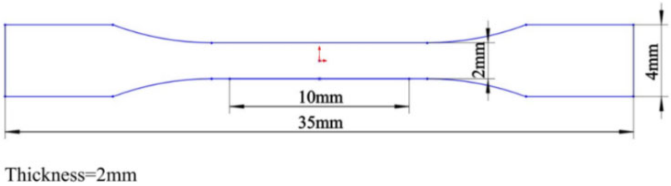

Figure S4. Schematic diagram of 1BB-type sample for the tensile test.
